# On the Use of Gene Ontology Annotations to Assess Functional Similarity among Orthologs and Paralogs: A Short Report

**DOI:** 10.1371/journal.pcbi.1002386

**Published:** 2012-02-16

**Authors:** Paul D. Thomas, Valerie Wood, Christopher J. Mungall, Suzanna E. Lewis, Judith A. Blake, on behalf of the Gene Ontology Consortium

**Affiliations:** 1 Division of Bioinformatics, Department of Preventive Medicine, University of Southern California, Los Angeles, California, United States of America; 2 Cambridge Systems Biology Centre and Department of Biochemistry, University of Cambridge, Cambridge, United Kingdom; 3 Genomics Division, Lawrence Berkeley National Laboratory, Berkeley, California, United States of America; 4 Bioinformatics and Computational Biology, The Jackson Laboratory, Bar Harbor, Maine, United States of America; University of California San Diego, United States of America

## Abstract

A recent paper (Nehrt *et al.*, PLoS Comput. Biol. 7:e1002073, 2011) has proposed a metric for the “functional similarity” between two genes that uses only the Gene Ontology (GO) annotations directly derived from published experimental results. Applying this metric, the authors concluded that paralogous genes *within* the mouse genome or the human genome are more functionally similar on average than orthologous genes *between* these genomes, an unexpected result with broad implications if true. We suggest, based on both theoretical and empirical considerations, that this proposed metric should not be interpreted as a functional similarity, and therefore cannot be used to support any conclusions about the “ortholog conjecture” (or, more properly, the “ortholog functional conservation hypothesis”). First, we reexamine the case studies presented by Nehrt *et al.* as examples of orthologs with divergent functions, and come to a very different conclusion: they actually exemplify how GO annotations for orthologous genes provide complementary information about conserved biological functions. We then show that there is a global ascertainment bias in the experiment-based GO annotations for human and mouse genes: particular types of experiments tend to be performed in different model organisms. We conclude that the reported statistical differences in annotations between pairs of orthologous genes do not reflect differences in biological function, but rather complementarity in experimental approaches. Our results underscore two general considerations for researchers proposing novel types of analysis based on the GO: 1) that GO annotations are often incomplete, potentially in a biased manner, and subject to an “open world assumption” (absence of an annotation does not imply absence of a function), and 2) that conclusions drawn from a novel, large-scale GO analysis should whenever possible be supported by careful, in-depth examination of examples, to help ensure the conclusions have a justifiable biological basis.

## Author Summary

Understanding gene function—how individual genes contribute to the biology of an organism at the molecular, cellular and organism levels—is one of the primary aims of biomedical research. It has been a longstanding tenet of model organism research that experimental knowledge obtained in one organism is often applicable to other organisms, particularly if the organisms share the relevant genes because they inherited them from their common ancestor. Nevertheless this tenet is, like any hypothesis, not beyond question. A recent paper has termed this hypothesis a “conjecture,” and performed a statistical analysis, the results of which were interpreted as evidence against the hypothesis. This statistical analysis relied on a computational representation of gene function, the Gene Ontology (GO). As representatives of the international consortium that produces the GO, we show how the apparent evidence against the “ortholog conjecture” can be better explained as an artifact of how molecular biology knowledge is accumulated. In short, a complementarity between knowledge obtained in mouse and human experimental systems was incorrectly interpreted as a disagreement. We discuss the proper interpretation of GO annotations and potential sources of bias, with an eye toward enhancing the informed use of the GO by the scientific community.

## Introduction

The Gene Ontology (GO) Consortium has, over the last 10 years, revolutionized the use of structured, controlled vocabularies in biology, and provides GO annotations of gene products that describe biological function from the molecular to organism level [1], [2]. During this time, the biocuration community, and in particular the curators associated with the major model organism databases (MODs), have contributed tens of thousands of GO annotations—associations between a specific gene or gene product and a term in the GO—based on experimental results reported in the biomedical literature. As this corpus of experimental annotations has grown, it has become increasingly powerful to mine the annotations within the context of the ontology structure not only to generate biological hypotheses but also to examine precepts of comparative biology. In a recent publication Nehrt *et al.* [3] used these experimentally-derived GO annotations to test the hypothesis that orthologous genes (separated by a speciation event) have more closely related functions than paralogous genes (separated by a gene duplication event). Here we discuss the applicability of GO annotations for their analysis, issues that impact the interpretation of the results they report, and some overall guidelines that should govern use of functional annotations in computational analysis. The Nehrt *et al.* paper highlights some potential pitfalls of using GO annotations without considered evaluation of the sources and semantics of these annotations [4].

In brief, the “ortholog conjecture” derives from a simple observation of genomic evolution: during evolution, genomes have often expanded via intra-genome copying of genomic regions (a process called “gene duplication”), and there are many documented cases in which one or more of the duplicates either adopted a new or modified function (“neofunctionalization”) or lost a function (“subfunctionalization”), resulting in duplicated genes with functions that differ to some degree [5]. These duplicates are referred to as “paralogs,” whether they are from the same genome (e.g. human hemoglobin vs. human myoglobin) or different genomes (e.g. human hemoglobin vs. mouse myoglobin) [6]. “Orthologs,” on the other hand, occur only in different genomes because they are separated by a speciation event (e.g. human myoglobin vs. mouse myoglobin) [6]. Because of the apparent importance of gene duplication in generating genes with novel or modified functions, it is generally assumed that orthologs tend, on average, to share a greater functional similarity than paralogs, the so-called “ortholog conjecture.” This hypothesis has been questioned [7].

Nehrt *et al.* claim to perform the first large-scale test of this hypothesis. The primary evidence the authors use to draw their conclusions is a score based on the normalized intersection of the experimentally-supported Gene Ontology annotations for different pairs of genes. The authors interpret the score as representative of functional similarity. We contend that the score is more accurately described as annotation congruence. These two interpretations are very different: functional similarity refers to similarity in the *actual biological function* of two gene products, while annotation congruence refers to agreement in the representation of the functions that have been *experimentally demonstrated so far* for two gene products. If our experimental knowledge of biological function were complete, and adequately represented by GO annotations, these would be equivalent. Unfortunately this is not yet the case in general. It is very important to note that GO annotations are subject to an “open world assumption”, i.e. *absence of a GO annotation does not mean that a function is absent from a particular gene product*. Even the limited knowledge that we *do* have about biological function is not yet completely represented by GO annotations, due to limitations of time and resources. Perhaps most importantly for this discussion, different model organisms are used to study different aspects of biology using different assays, and so the annotation of orthologs in different species will reflect these systematic differences in experimental systems and outcomes. In fact, complementarity with other established systems is a key factor in the development of different model organism experimental systems. As a result of these and other considerations, we suggest that the authors, rather than testing the “ortholog conjecture,” instead tested an “unbiased annotation conjecture.” Similar suggestions have been made in post-publication review forums (http://f1000.com/12462957?key=5g7rjmt7xzv2y32) and blogs (http://phylogenomics.blogspot.com/2011/09/special-guest-post-discussion.html), but not yet in the peer-reviewed literature.

As Nehrt *et al.* describe, it would indeed be contrary to expectations if paralogous genes in humans or mice were functionally more similar than orthologous genes between these species. This would not only challenge the so-called “ortholog conjecture”: it would challenge the longstanding research programs in model systems and comparative biology, and even the tenets of current evolutionary theory with its emphasis on inheritance and divergence from a common ancestor. Surprisingly, then, the rejection of the “ortholog conjecture” by Nehrt *et al.* is based almost entirely on statistical analysis of existing GO annotations, with no in-depth analysis of specific examples. In particular, the section entitled “Case studies” provides no citation of experimental evidence for the authors' claims, thus complicating overall evaluations. Here, we examine these specific cases, and find no evidence for the conclusion that within-species paralogs are more functionally similar than orthologs. Instead, we suggest that the statistical bias observed by Nehrt *et al.* is better explained by a bias in annotations arising at least in part because research programs in human and mouse experimental systems tend to discover aspects of orthologous gene function that are *complementary* rather than conflicting.

## Results

Nehrt *et al.* examined two different case studies that showed particularly large increases in annotation similarity between paralogs as compared to orthologs. In order to support the interpretation of annotation similarity as functional similarity, the case studies would need to present evidence of true biological similarity rather than evidence of annotation similarity. We therefore examined these case studies in more detail.

### Case 1: MAP4K2

Mitogen activated protein kinase kinase kinase kinases (MAP4K) are protein kinases that participate in the MAP kinase signal transduction cascade [8]. The authors state that an “example of a violation of the ortholog conjecture is… *MAP4K2*…While the human *hMAP4K2* shares 94% sequence identity with its ortholog in mouse, their functional similarity is only 5% (45 annotated terms in human, 13 in mouse). In contrast, its functional similarity with its own outparalogs was 69% on average, including 82% similarity with *hMAP4K3*, a within-species outparalog.” The GO biological process annotations for human *MAP4K2*, mouse *Map4k2* and human *MAP4K3* are shown in Table 1. Both human *MAP4K2* and human *MAP4K3* are annotated with *intracellular protein kinase cascade* (GO:0007243) and *protein phosphorylation* (GO:0006468), while mouse *Map4k2* is only annotated with *vesicle targeting* (GO:0006903). So the finding that the annotation congruence for *MAP4K2* and *MAP4K3* in humans (paralogs) is greater than for human *MAP4K2* and mouse *Map4k2* (orthologs) is correct.

**Table 1:** Experimentally-supported GO annotations for *MAP4K2* and *MAP4K3* genes in human, and *Map4k2* gene in mouse.

GO biological process	*MAP4K2*	*Map4k2*	*MAP4K3*
GO:0065007 biological regulation	x		x
GO:0050789 regulation of biological process	x		x
GO:0060255 regulation of macromolecule metabolic process	x		
GO:0080090 regulation of primary metabolic process	x		
GO:0051716 cellular response to stimulus	x		x
GO:0031323 regulation of cellular metabolic process	x		
GO:0050794 regulation of cellular process	x		
GO:0019222 regulation of metabolic process	x		
GO:0065009 regulation of molecular function	x		
GO:0051174 regulation of phosphorus metabolic process	x		
GO:0051246 regulation of protein metabolic process	x		
GO:0044093 positive regulation of molecular function	x		
GO:0050790 regulation of catalytic activity	x		
GO:0032268 regulation of cellular protein metabolic process	x		
GO:0019220 regulation of phosphate metabolic process	x		
GO:0009987 cellular process	x	x	x
GO:0035556 intracellular signal transduction	x		x
GO:0008152 metabolic process	x		x
GO:0043085 positive regulation of catalytic activity	x		
GO:0042325 regulation of phosphorylation	x		
GO:0031399 regulation of protein modification process	x		
GO:0051338 regulation of transferase activity	x		
GO:0006950 response to stress	x		xx
GO:0007154 cell communication	x		x
GO:0044237 cellular metabolic process	x		x
GO:0033554 cellular response to stress	x		
GO:0007243 intracellular protein kinase cascade	x		xx
GO:0051347 positive regulation of transferase activity	x		
GO:0044238 primary metabolic process	x		x
GO:0043549 regulation of kinase activity	x		
GO:0001932 regulation of protein phosphorylation	x		
GO:0080134 regulation of response to stress	x		
GO:0050896 response to stimulus	x		
GO:0023052 signaling	x		x
GO:0044260 cellular macromolecule metabolic process	x		
GO:0043170 macromolecule metabolic process	x		
GO:0000165 MAPKKK cascade	x		
GO:0006793 phosphorus metabolic process	x		x
GO:0033674 positive regulation of kinase activity	x		
GO:0019538 protein metabolic process	x		x
GO:0080135 regulation of cellular response to stress	x		
GO:0010627 regulation of intracellular protein kinase cascade	x		
GO:0045859 regulation of protein kinase activity	x		
GO:0048583 regulation of response to stimulus	x		
GO:0023051 regulation of signaling	x		
GO:0007165 signal transduction	x		x
GO:0031098 stress-activated protein kinase signaling cascade	x		
GO:0044267 cellular protein metabolic process	x		x
GO:0007254 JNK cascade	x		
GO:0043412 macromolecule modification	x		x
GO:0006796 phosphate-containing compound metabolic process	x		x
GO:0045860 positive regulation of protein kinase activity	x		
GO:0043408 regulation of MAPKKK cascade	x		
GO:0071900 regulation of protein serine/threonine kinase activity	x		
GO:0009966 regulation of signal transduction	x		
GO:0070302 regulation of stress-activated protein kinase signaling cascade	x		
GO:0016310 phosphorylation	x		x
GO:0071902 positive regulation of protein serine/threonine kinase activity	x		
GO:0006464 protein modification process	x		x
GO:0046328 regulation of JNK cascade	x		
GO:0043405 regulation of MAP kinase activity	x		
GO:0043406 positive regulation of MAP kinase activity	x		
GO:0006468 protein phosphorylation	x		xx
GO:0043506 regulation of JUN kinase activity	x		
GO:0000187 activation of MAPK activity	x		
GO:0043507 positive regulation of JUN kinase activity	x		
GO:0007257 activation of JUN kinase activity	xx		
GO:0051641 cellular localization		x	
GO:0051179 localization		x	
GO:0051234 establishment of localization		x	
GO:0051640 organelle localization		x	
GO:0051649 establishment of localization in cell		x	
GO:0051656 establishment of organelle localization		x	
GO:0006810 transport		x	
GO:0051648 vesicle localization		x	
GO:0051650 establishment of vesicle localization		x	
GO:0016192 vesicle-mediated transport		xx	

‘x’ means inferred annotation (direct annotation by curator was to a child term); ‘xx’ means direct annotation. The “functional similarity” (actually an annotation congruence score) as defined by Nehrt *et al.* includes all terms, both inferred and direct.

However, decreased annotation congruence can be explained more easily in terms of annotation incompleteness (arising from incompleteness in actual experimental results) and complementarity rather than functional differences between orthologs. MAP4Ks are upstream of MAP3Ks in the mitogen-activated protein kinase (MAPK) cascade, and both *MAP4K2* and *MAP4K3* are known in humans to participate specifically in the JNK (c-Jun N-terminal kinase) cascade, one of four different known MAPK pathway variants [8]. Thus, from a functional standpoint, it is generally accepted that human *MAP4K2* and *MAP4K3*, and mouse *Map4K2* can all participate in an intracellular protein kinase cascade. However, from a GO annotation standpoint, only human *MAP4K2* and *MAP4K3* have been experimentally characterized as participating in an intracellular protein kinase cascade. Mouse *Map4K2*, the ortholog of human *MAP4K2*, has apparently not been characterized at the molecular level, though there are several reported effects of mouse mutants lacking *Map4k2*, including an effect on vesicle targeting. This lack of experimental characterization cannot, however, be taken as evidence that *Map4k2* differs from its human ortholog in that it does not participate in MAPK signaling. On the contrary, a molecular link between the JNK cascade and vesicle targeting (through the conserved JNK-interacting protein JIP-1) has been established in *Drosophila* [9], suggesting a mechanism by which mouse *Map4k2* (and likely its human ortholog) may affect vesicle targeting *through* MAPK signaling. In summary, the different annotations for mouse and human orthologs of *MAP4K2* do not constitute evidence that the orthologous genes have different functions; a more likely explanation is that they are instead providing complementary information about a conserved biological system, representing the current, incomplete, state of experimentation results.

### Case 2: Nuclear receptors

Nuclear receptors are transcription factors, influencing transcription of specific target genes, that are activated by binding a specific ligand. The authors find that, in this family, “a paralog was more functionally similar than the ortholog for the majority of the targets, and the specific paralog with the highest functional similarity was most often an outparalog in the same species.” The biological functions of nuclear receptors are known to be highly dependent upon their biological ligands, and the evolution of ligand specificity has been studied for some members of this family [10], [11]. The authors provide no specific comparisons in this family, we therefore selected an example to illustrate that quantitative differences in annotation congruence score as defined in this paper may not be functionally meaningful. The thyroid hormone receptor alpha (*THRA* in human, *Thra* in mouse) gene product binds thyroid hormone, a tyrosine-based hormone, and has effects on tissue growth, differentiation and metabolism [12]. The estrogen receptor alpha (*Esr1* in mouse) gene product binds the steroid hormone estrogen (the primary female hormone in mammals), and has physiological effects ranging from reproduction to cognition [13]. The thyroid receptor and estrogen receptor bind chemically different ligands, and activate very different sets of target genes. There is no known biological evidence that the mouse thyroid receptor is more similar in its actual biological function to its paralog *Esr1*, than to its human ortholog *THRA*. Indeed, such a convergence in function between paralogs would be a revolutionary finding.

Yet the molecular function annotation congruence for mouse *Thra* is greater with mouse *Esr1* than with human *THRA* (Table 2). Is there any evidence that mouse *Thra* is actually more similar in function to its paralog *Esr1* than to its human ortholog, even in the GO annotations? There is not: the GO annotations are correct, if incomplete. The observed greater annotation similarity for *Thra*-*Esr1* is driven largely by the greater *specificity* in the annotations of human *THRA* as compared to either mouse gene. Both mouse genes are annotated with 1) *protein binding*, and 2) *ligand-activated sequence-specific DNA binding RNA polymerase II transcription factor activity*, while *THRA* is annotated with 1) *TBP-class protein binding* and 2) *thyroid hormone receptor activity*. *TBP-class protein binding* is a subclass of *protein binding*, while *thyroid hormone receptor activity* is a subclass of *ligand-activated sequence-specific DNA binding RNA polymerase II transcription factor activity*. It is important to consider the semantics of a non-specific GO annotation: an annotation of mouse *Thra* as possessing *ligand-activated sequence-specific DNA binding RNA polymerase II transcription factor activity* means that the gene product functions as a nuclear receptor for *some* (unspecified) ligand, which of course does not preclude that the ligand is thyroid hormone. Thus differences in annotation specificity, a form of annotation incompleteness, cannot generally be interpreted as differences in actual biological function. Differences in annotation specificity, even for similar experiments, may arise for non-biological reasons such as variability in annotation processes between different curation groups (note that most GO annotations for human genes are made by GOA [14] while for mouse genes most are made by MGI [15]), differences in the experimental systems employed in different research laboratories, and the differences in availability of terms in the ontology at the time of annotation.

**Table 2:** Experimentally-supported GO annotations for *Thra* and *Esr1* genes in mouse, and *THRA* gene in human.

GO molecular function	*Thra*	*THRA*	*Esr1*
GO:0060089 molecular transducer activity	x	x	x
GO:0001071 nucleic acid binding transcription factor activity	x	x	x
GO:0004872 receptor activity	x	x	x
GO:0003700 sequence-specific DNA binding transcription factor activity	x	x	x
GO:0004871 signal transducer activity	x	x	x
GO:0000981 sequence-specific DNA binding RNA polymerase II transcription factor activity	x	x	x
GO:0038023 signaling receptor activity	x	x	x
GO:0004879 ligand-activated sequence-specific DNA binding RNA polymerase II transcription factor activity	xx	x	xx
GO:0004887 thyroid hormone receptor activity		xx	
GO:0005488 binding	x	x	x
GO:0005515 protein binding	xx	x	xx
GO:0032403 protein complex binding	xx		
GO:0008134 transcription factor binding		x	xx
GO:0017025 TBP-class protein binding		xx	
GO:0019904 protein domain specific binding		xx	
GO:0003676 nucleic acid binding	x	x	
GO:0003723 RNA binding	x		
GO:0003727 single-stranded RNA binding	x		
GO:0002153 steroid receptor RNA activator RNA binding	xx		
GO:0042562 hormone binding		x	
GO:0070324 thyroid hormone binding		xx	
GO:0003677 DNA binding		x	
GO:0001067 regulatory region nucleic acid binding		x	
GO:0000975 regulatory region DNA binding		xx	
GO:0003682 chromatin binding			xx

‘x’ means inferred annotation (direct annotation by curator was to a child term); ‘xx’ means direct annotation. The “functional similarity” (actually an annotation congruence score) as defined by Nehrt *et al.* includes all terms, both inferred and direct.

### GO annotations are incomplete, and biased by differences in experimental systems

Nevertheless, assuming that the annotation similarity scores are calculated correctly, the statistical differences reported by Nehrt *et al.* between orthologs and paralogs are significant. However if, as suggested above, the differences are not biological in origin, is there an alternative interpretation? The authors observed that the greatest differences in annotation similarity scores occur between two groups: 1) inparalogs/within-species outparalogs, versus 2) orthologs/between-species outparalogs. In short, within-species comparisons yielded greater annotation similarity scores on average than between-species comparisons. The authors claim that “the sparsity of annotation… is unlikely to affect comparisons between classes of homologs,” but this claim is essential for their interpretations and requires supporting evidence. As shown in the examples above, annotation incompleteness can result in annotation differences even in the absence of functional differences. We reasoned that the bias uncovered by Nehrt *et al.*, in which within-species comparisons showed greater annotation similarity than between-species comparisons, would arise if GO annotations for mouse genes *in general*—not just for paralogous genes—are more similar to each other than to human GO annotations, and vice versa.

To test this alternative explanation, we compared the set of *all* human experimental annotations to the set of *all* mouse experimental annotations in the GO database. Table 3 lists several examples of molecular functions and biological processes that are very unequally represented in the annotations for one species relative to the other. For molecular function, human annotations are enriched in protein binding and some enzymatic functions, while mouse annotations are enriched in transcription factors and ion channels. In agreement with Nehrt *et al.*'s results (but contrary to their interpretation), biological process annotations are even more biased, with mouse being enriched for organism-level processes including development and cell differentiation, and human for cellular biochemical-level processes such as protein modification and molecular catabolism. These differences in overrepresented functional classes are very unlikely to reflect actual functional differences between human and mouse orthologs; rather they reflect biases both in the kinds of experiments that are performed in that organism, and in the curation process (e.g. which published papers are prioritized for annotation by a given curation group). Some of the most significant biases can be explained by the fact that mouse is used in genetics experiments to probe organism level processes that cannot be approached experimentally in humans, while many of the experiments in human systems are performed on isolated cells and proteins.

**Table 3:** GO annotation classes overrepresented in mouse compared to human, or vice versa.

Aspect	GO ID	GO term	# mouse annotations	# human annotations	P-value
molecular function	GO:0005515	protein binding	6151	12318	<10^−100^
molecular function	GO:0016462	pyrophosphatase activity	109	240	<10^−50^
molecular function	GO:0003682	chromatin binding	204	68	<10^−30^
molecular function	GO:0005261	cation channel activity	187	75	<10^−20^
molecular function	GO:0003700	sequence-specific DNA binding transcription factor activity	427	252	<10^−10^
biological process	GO:0032502	developmental process	22114	3197	<10^−100^
biological process	GO:0032501	multicellular organismal process	15070	2987	<10^−100^
biological process	GO:0030154	cell differentiation	5390	1035	<10^−100^
biological process	GO:0043412	macromolecule modification	1438	2277	<10^−100^
biological process	GO:0044248	cellular catabolic process	523	904	<10^−100^
biological process	GO:0051276	chromosome organization	338	634	<10^−100^

P-value is calculated using hypergeometric distribution without Bonferroni correction.

## Discussion

We have shown that the interpretation of Nehrt *et al.*'s metric of GO annotation congruence as functional similarity is problematic, and therefore it cannot be used to draw valid conclusions about the ortholog functional conservation hypothesis. From a theoretical standpoint, the semantics of GO annotations must be interpreted using an “open world assumption” in which absence of an annotation does not mean absence of a function (a true negative). Thus, lack of annotation congruence may simply be due to false negatives: incompleteness either in the state of our experiment-derived knowledge of a particular gene's function, or in representing that knowledge as GO annotations. From an empirical standpoint, we demonstrate that the bias noted by Nehrt *et al.* between different classes of homologous gene in human and mouse, is likely to be reflecting a global bias over *all* human and mouse genes. This global bias is consistent with the common use of mouse as a genetic system for probing system-level processes via observed phenotypes, and of the use of human cell lines for probing cellular-level processes. It may also reflect a tendency for researchers not to “repeat” a particular experiment that has already been carried out in a closely related organism.

We note that Nehrt *et al.* did attempt to address potential sources of bias in GO annotations, though they apparently missed a major contributor as discussed above. The authors' observation that there are “preferences toward the same annotation when multiple homologs were functionally annotated in the same article: functional similarity went up 0.1–0.3 across orthologs and paralogs for both Biological Process and Molecular Function” supports the “biased annotation conjecture” interpretation we propose here. We would also expect annotation congruence to increase accordingly if homolog annotations were derived from research groups and co-authors addressing the same biological questions, or for annotations made during the same time period, when they would be constrained by the availability of similar GO terms.

Nevertheless, whenever a novel type of GO-based statistical analysis is presented, a manual review of key examples or case studies should be considered as an important component of validating its biological implications. GO-based analysis can be an excellent way to generate biological hypotheses, but in order to draw defensible conclusions, it is important to verify actual biological examples, particularly if analyses may be affected by global differences between the sets of annotations being compared. Between-species comparisons based on different annotation sources (i.e. organisms), are particularly sensitive to subtle differences in annotation and experimental testing bias. Users of GO should ensure that they test for, and adjust for, potential biases prior to interpretation. Our re-analysis of the case studies presented by Nehrt *et al.* confirmed a greater annotation congruence between paralogs as compared to orthologs, but showed that this difference is due to incomplete and complementary annotations, and not to functional divergence among orthologs or convergence among paralogs. This in-depth analysis suggested possible types of bias that we explored with further interrogation of biological knowledge and statistical analysis.

If the annotation congruence is not appropriate, are there alternative ways in which GO annotations might be used to test the ortholog functional conservation hypothesis? One way that functional differences between orthologs and paralogs could be addressed using GO would be to consider homologs for which similar experiments had been performed, and where negative results were captured as negative GO annotations (using the “NOT” qualifier) to indicate the absence of functionality. We note that GO curators have already made numerous negative annotations—though these are still very incomplete—often where a particular function was suspected/expected for a gene (one possible reason being that it was found for an ortholog) but shown not to be present. Two examples of orthologs with divergent functions are *SUV3* (*Saccharomyces cerevisiae*)/*rpm2* (*Schizosaccharomyces pombe*) and *MGT1* (*S. cerevisiae*)/*atl1* (*S. pombe*). In these cases, the gene product in *S. pombe* has been demonstrated to lack a function found in the *S. cerevisiae* ortholog, and this has been captured with negative annotations for the *S. pombe* genes [16], [17]. To date, negative GO annotations are relatively rare and probably insufficient to refute or support the ortholog functional conservation hypothesis in general, though a detailed and careful analysis might be interesting. Indeed, several functional differences between orthologous genes in humans and mice have been documented [18], but it is unclear how prevalent such cases will prove to be as more experimental data accumulate.

We applaud the use of the Gene Ontology resources in new and creative ways. At the same time, we strongly encourage careful consideration of the interpretations of such uses. Do they reflect actual biological insights, or are they in fact due to inherent biases in annotation and or the experimental data or systems available? This phenomenon is certainly not limited to GO analyses. The process of knowledge representation of any kind will always introduce issues that must be properly considered in meta-analyses. We strongly and actively encourage researchers to contact us when proposing a novel type of GO-based analysis, to ensure appropriate interpretation and use of the GO.

## Methods

Term overrepresentation analysis (Table 3) was performed on the sets of human and mouse annotations from the 2011-09-10 release of the GO database, using the cumulative hypergeometric probability distribution in Microsoft Excel. Only annotations with the following evidence codes were considered: EXP, IPI, IDA, IMP, IGI, IEP (http://www.geneontology.org/GO.evidence.shtml). For the *MAP4K2* and nuclear receptor examples (Tables 1 and 2), GO annotations (same evidence codes as above) were retrieved using AMIGO (http://www.geneontology.org) on 2011-11-29.
